# High-dose versus standard-dose vitamin D supplementation in older adults with COVID-19 (COVIT-TRIAL): A multicenter, open-label, randomized controlled superiority trial

**DOI:** 10.1371/journal.pmed.1003999

**Published:** 2022-05-31

**Authors:** Cédric Annweiler, Mélinda Beaudenon, Jennifer Gautier, Justine Gonsard, Sophie Boucher, Guillaume Chapelet, Astrid Darsonval, Bertrand Fougère, Olivier Guérin, Marjorie Houvet, Pierre Ménager, Claire Roubaud-Baudron, Achille Tchalla, Jean-Claude Souberbielle, Jérémie Riou, Elsa Parot-Schinkel, Thomas Célarier, on behalf of the COVIT-TRIAL study group

**Affiliations:** 1 Department of Geriatric Medicine and Memory Clinic, Research Centre on Autonomy and Longevity, University Hospital, Angers, France; UNIV Angers, UPRES EA 4638, University of Angers, Angers, France; 2 Delegation for Clinical Research and Innovation, Angers University Hospital, Angers, France; 3 Department of ENT Head and Neck Surgery, University Hospital, Angers, France; MitoLab Team Mitochondrial Medecine Research Centre, MitoVasc Intitute, UNIV Angers, CNRS UMR6015, INSERM U1083, University of Angers, Angers, France; 4 Department of Geriatric Medicine and Memory Clinic, Research Centre, University Hospital, Nantes, France; Inserm UMR1235, Nantes Université, Nantes, France; 5 Department of Pharmacy, Angers University Hospital, Angers, France; 6 Division of Geriatric Medicine, Tours University Hospital, Tours, France; Education, Ethics, Health (EA 7505), Tours University, Tours, France; 7 Université Côte d’Azur, Centre Hospitalier Universitaire de Nice, Service de Médecine Gériatrique et Thérapeutique, Nice, France; Université Côte d’Azur, CNRS UMR 7284/INSERM U108, Institute for Research on Cancer and Aging Nice (IRCAN), Faculté de Médecine, Nice, France; 8 Department of Geriatric Medicine, General Hospital, Saumur, France; 9 Department of Geriatric Medicine, General Hospital, Le Mans, France; 10 Pôle de Gérontologie Clinique, CHU Bordeaux, Bordeaux, France; INSERM 1053, Univ. Bordeaux, Bordeaux, France; 11 Unité de Recherche Clinique et d’Innovation (URCI) de Gérontologie, Pôle HU Gérontologie Clinique, CHU de Limoges, France; Université de Limoges, IFR GEIST, Laboratoire UR Vie-Santé (Aging and Digital health Lab) Faculté de Medicine de Limoges, Limoges, France; 12 Service des Explorations Fonctionnelles, Hôpital Necker-Enfants Malades, AP-HP, Paris, France; 13 Department of Clinical Gerontology, University Hospital of Saint-Etienne, Saint-Etienne, France; Chaire Santé des Ainés, University of Jean Monnet, Saint-Etienne, France; Leiden University Medical Center, NETHERLANDS

## Abstract

**Background:**

Vitamin D supplementation has been proposed as a treatment for Coronavirus Disease 2019 (COVID-19) based on experimental data and data from small and uncontrolled observational studies. The COvid19 and VITamin d TRIAL (COVIT-TRIAL) study was conducted to test whether a single oral high dose of cholecalciferol (vitamin D3) administered within 72 hours after the diagnosis of COVID-19 improves, compared to standard-dose cholecalciferol, the 14-day overall survival among at-risk older adults infected with Severe Acute Respiratory Syndrome Coronavirus 2 (SARS-CoV-2).

**Methods and findings:**

This multicenter, randomized, controlled, open-label, superiority trial involved collaboration of 9 medical centers in France. Patients admitted to the hospital units or living in nursing homes adjacent to the investigator centers were eligible if they were ≥65 years, had SARS-CoV-2 infection of less than 3 days, and at least 1 COVID-19 worsening risk factor (among age ≥75 years, SpO2 ≤94%, or PaO_2_/FiO_2_ ≤300 mm Hg). Main noninclusion criteria were organ failure requiring ICU, SpO2 ≤92% despite 5 L/min oxygen, life expectancy <3 months, vitamin D supplementation >800 IU/day during the preceding month, and contraindications to vitamin D supplements. Eligible and consenting patients were randomly allocated to either a single oral high-dose (400,000 IU) or standard-dose (50,000 IU) cholecalciferol administered under medical supervision within 72 hours after the diagnosis of COVID-19. Participants and local study staff were not masked to the allocated treatment, but the Steering Committee and the Data and Safety Monitoring Board were masked to the randomization group and outcome data during the trial. The primary outcome was 14-day overall mortality. Between April 15 and December 17, 2020, of 1,207 patients who were assessed for eligibility in the COVIT-TRIAL study, 254 met eligibility criteria and formed the intention-to-treat population. The median age was 88 (IQR, 82 to 92) years, and 148 patients (58%) were women. Overall, 8 (6%) of 127 patients allocated to high-dose cholecalciferol, and 14 (11%) of 127 patients allocated to standard-dose cholecalciferol died within 14 days (adjusted hazard ratio = 0.39 [95% confidence interval [CI], 0.16 to 0.99], *P* = 0.049, after controlling for randomization strata [i.e., age, oxygen requirement, hospitalization, use of antibiotics, anti-infective drugs, and/or corticosteroids] and baseline imbalances in important prognostic factors [i.e., sex, ongoing cancers, profuse diarrhea, and delirium at baseline]). The number needed to treat for one person to benefit (NNTB) was 21 [NNTB 9 to ∞ to number needed to treat for one person to harm (NNTH) 46]. Apparent benefits were also found on 14-day mortality due to COVID-19 (7 (6%) deaths in high-dose group and 14 (11%) deaths in standard-dose group; adjusted hazard ratio = 0.33 [95% CI, 0.12 to 0.86], *P* = 0.02). The protective effect of the single oral high-dose administration was not sustained at 28 days (19 (15%) deaths in high-dose group and 21 (17%) deaths in standard-dose group; adjusted hazard ratio = 0.70 [95% CI, 0.36 to 1.36], *P* = 0.29). High-dose cholecalciferol did not result in more frequent adverse effects compared to the standard dose. The open-label design and limited study power are the main limitations of the study.

**Conclusions:**

In this randomized controlled trial (RCT), we observed that the early administration of high-dose versus standard-dose vitamin D3 to at-risk older patients with COVID-19 improved overall mortality at day 14. The effect was no longer observed after 28 days.

**Trial registration:**

ClinicalTrials.gov NCT04344041.

## Abstract 2

In a randomized trial, Cedric Annweiler and colleagues evaluate whether a single high dose of vitamin D3 improves survival among older adults in France with SARS-CoV-2 infection.

## Author summary

**Why was this study done?:**

- Preclinical data suggest that vitamin D may attenuate the effects of Severe Acute Respiratory Syndrome Coronavirus 2 (SARS-CoV-2) by exerting anti-inflammatory effects and by regulating the renin–angiotensin system, thus potentially preventing the cytokine storm and its lethal consequences.
- Observational studies have reported that, while accounting for confounders, participants with lower 25-hydroxyvitamin D (25(OH)D) concentrations were more likely to progress to severe forms of Coronavirus Disease 2019 (COVID-19) and to die from COVID-19.
- Observational studies have also found that vitamin D3 supplementation prior to or during COVID-19 was associated with improved survival in older adults with COVID-19.
- Clinical trials to evaluate the impact on survival of high-dose vitamin D supplementation in COVID-19 patients have yet not been performed.

**What did the researchers do and find?:**

- In this multicenter, open-label, randomized controlled trial (RCT), we randomly assigned 254 at-risk older adults with COVID-19 to a single oral high-dose (400,000 IU) or standard-dose (50,000 IU) vitamin D3 administered within 72 hours after the diagnosis of COVID-19, with 14-day survival as the primary outcome.
- We found that the early administration of 400,000 IU versus 50,000 IU vitamin D3 to at-risk older patients with COVID-19 was associated with reduced overall mortality at day 14. The protective effect was not sustained at 28 days.
- High-dose cholecalciferol did not result in more frequent adverse effects compared to the standard dose.

**What do these findings mean?:**

- In the absence of toxicity and given the benefits of high-dose vitamin D found on 14-day mortality, a combination therapy with both standard treatments for COVID-19 and high doses of vitamin D3 may be proposed to at-risk older patients with COVID-19 within the first hours of the infection.
- The lack of protection after 28 days should encourage examination of the possible benefit of a continuous daily (or weekly) vitamin D supplementation following the initial loading dose.
- Our study was not designed to determine whether vitamin D supplementation helps with prevention of SARS-CoV-2 infection.

## Introduction

The Coronavirus Disease 2019 (COVID-19) caused by the Severe Acute Respiratory Syndrome Coronavirus 2 (SARS-CoV-2) spreads worldwide, affecting millions of people and causing hundreds of thousands deaths, mostly in older adults. The rapid development and authorization of vaccines against COVID-19 has given solid hope to end the pandemic in the near term [1]. However, the challenges to organize global vaccination programs and the emergence of immune escape variants justify continuing to explore additional drugs contributing to the prevention of severe and fatal forms of COVID-19.

An *in silico* study has identified vitamin D among the 3 molecules most likely to attenuate the effects of SARS-CoV-2 through its effects on genes expression [2]. Vitamin D is known to contribute to the defenses of mucous membranes by stimulating the secretion of antimicrobial peptides [3]. Vitamin D also exerts an anti-inflammatory action by stimulating the synthesis of anti-inflammatory cytokines while inhibiting the synthesis of pro-inflammatory cytokines [3] and inhibits renin secretion [4,5], potentially preventing the adverse effects of the activation of the renin–angiotensin system that follows the angiotensin converting enzyme 2 (ACE2) down-regulation secondary to the attachment of SARS-CoV-2. These effects may help preventing the cytokine storm that contributes to the severe forms of COVID-19 [6]. Several observational studies have confirmed that, while accounting for potential confounders, participants with lower serum 25-hydroxyvitamin D (25(OH)D) concentrations were more likely to progress to severe forms of COVID-19 [7], to resort to noninvasive ventilation [8], and, ultimately, to die from COVID-19 [9]. Vitamin D3 supplementation prior to COVID-19 [10,11] and during COVID-19 [12–14] was associated with improved survival in older adults with COVID-19. The latter studies were yet limited by their observational design.

At the start of the first wave of the pandemic, we hypothesized in the COvid19 and VITamin d TRIAL (COVIT-TRIAL) study that high-dose vitamin D supplementation could improve survival in older adults infected with SARS-CoV-2. The COVIT-TRIAL study was conducted to test whether a single oral high dose of cholecalciferol (vitamin D3) administered within 72 hours after the diagnosis of COVID-19 improves, compared to standard-dose cholecalciferol, the 14-day overall survival among at-risk older adults infected with SARS-CoV-2.

## Methods

### Study design

This investigator-initiated, multicenter, open-label, parallel group, intent-to-treat, randomized controlled superiority clinical trial involved collaboration of 9 medical centers in France (Angers University Hospital, Bordeaux University Hospital, Le Mans Hospital, Limoges University Hospital, Nantes University Hospital, Nice University Hospital, Saumur Hospital, Saint-Etienne University Hospital, Tours University Hospital). Details of the trial protocol and statistical analysis plan have been published previously [15]. The trial was coordinated by the Department of Geriatric Medicine at the University Hospital of Angers, France. The trial was done in accordance with the principles of the International Conference on Harmonization–Good Clinical Practice guidelines and approved by the French “Sud-Est V Ethics Committee” (20.04.03.65603, Grenoble, France; ref20-ANGE-01) and the French National Agency for Medicines and Health Products Safety (ANSM). It was supervised by an independent data and safety monitoring board. All authors and contributors are listed in S1 Supplemental Appendix. The study is reported as per the Consolidated Standards of Reporting Trials (CONSORT) guideline (S1 CONSORT Checklist).

### Setting and participants

Older adults admitted to the hospital units or living in nursing homes adjacent to the investigator centers were eligible to participate if they were 65 years of age or older, if they had SARS-CoV-2 infection diagnosed within the preceding 3 days by a reverse transcription polymerase chain reaction (RT-PCR, 45 cycles) test and/or chest computed tomography (CT)-scan, if they had at least one of the following COVID-19 worsening risk factors: age≥75years, or peripheral capillary oxygen saturation (SpO2) ≤94% on room air, or partial pressure of oxygen in arterial blood/fraction of inspired oxygen (PaO_2_/FiO_2_) ratio ≤300 mm Hg, and if they were covered by or had the rights to medical care insurance. Noninclusion criteria were as follows: organ failure requiring admission to intensive care unit (ICU), SpO2 ≤ 92% despite oxygen therapy >5 L/min, life expectancy <3 months, any reason preventing follow-up at 28 days, vitamin D supplementation of more than 800 IU per day during the preceding month, contraindications to the use of vitamin D supplements (i.e., active granulomatosis [sarcoidosis, tuberculosis, and lymphoma], history of calcium lithiasis, known hypervitaminosis D or hypercalcemia, and known intolerance to vitamin D supplements), enrollment in another simultaneous randomized controlled trial (RCT), and deprivation of liberty by administrative or judicial decision. Baseline serum 25(OH)D concentration was not used for eligibility. Written informed consent was obtained from all eligible participants, or a legal representative if they were too unwell or unable to provide consent, or using an emergency inclusion procedure resorting to a postal informed consent form in the context of national lockdown, as appropriate.

### Randomization

Eligible participants were randomly assigned to receive a single oral dose of either 400,000 IU or 50,000 IU cholecalciferol (Mylan, 75008 Paris, France) on the day of inclusion. Treatment allocation was carried out according to a 1:1 ratio by means of dynamic randomization, with the use of a minimization algorithm, considering 6 criteria: worsening risk factor (i.e., age ≥75 years or oxygen dependency characterized by SpO2 ≤94% on room air or PaO2/FiO2 ≤300 mm Hg), COVID-19 diagnostic test (i.e., RT-PCR or chest CT scan), hospitalization, concomitant use of antibiotics and anti-infective drugs, concomitant use of corticosteroids, and recruiting center. To prevent predictability, a probability of 0.80 to assign treatment that minimized imbalance was used, and 26 patients (10%) were randomized before applying the algorithm. The randomization was established by the Department of Biostatistics and Methodology of the University Hospital of Angers, France, using a web-based system (Ennov Clinical).

### Procedures

Cholecalciferol supplement was taken under medical supervision on the day of inclusion, ideally during food intakes because this lipophilic vitamin is better absorbed with fat. As the appearance and number of drinking vials varied according to the assignment to high-dose (two 200,000 IU vials at once) and standard-dose vitamin D3 groups (one 50,000 IU vial), participants and local study staff were not masked to the allocated treatment. Moreover, since the serum 25(OH)D concentration measured at day 7 could give indications on the dose of cholecalciferol administered, data were not coded for the analyses. Both the Steering Committee and the Data and Safety Monitoring Board remained masked to the randomization group and outcome data during the trial.

Three follow-up visits were scheduled at 7, 14, and 28 days after the randomization. Information was recorded regarding the participants’ clinical signs, routine healthcare data, laboratory testing, receipt of other treatments and/or respiratory support for COVID-19, and vital status (including the adjudicated cause of death).

Blood samples from baseline (before vitamin D3 administration) and day 7 (±1 day) were obtained in the morning and thawed within 4 hours of sampling. Serums were analyzed locally at each site to measure changes in serum 25(OH)D concentration by chemiluminescent immunoassay (LIAISON XL, DiaSorin, Saluggia, Italy). Immunoassay kits recognize both vitamin D2 and D3. The intra- and interassay precisions are respectively 5.2% and 11.3% (range in normal adults aged 20 to 60 years, 75 to 310 nmol/L). Safety criteria were also assessed, including calcium and creatinine concentrations. Hypercalcemia at day 7 was defined as serum calcium levels above 2.65 mmol/L and severe kidney failure as an estimated glomerular filtration rate (Cockcroft formula) below 30 mL/min/1.73m^2^.

To maximize the ability of the trial to observe a treatment effect, participants were asked not to take outside-of-trial vitamin D supplements (including multivitamins) until 28 days after randomization in the standard-dose vitamin D3 group and until 45 days in the high-dose vitamin D3 group. Trial completion was defined as completion of 28 days or discontinuation of follow-up for any cause.

### Outcomes and follow-up

The primary outcome was overall mortality within 14 days after randomization. Secondary outcomes were overall mortality within 28 days after randomization, mortality due to COVID-19 at 14 and 28 days, and between-group comparison of safety. Data on vital status were available for all participants at day 14 and were missing at day 28 in one participant from the high-dose vitamin D3 group and in one participant from the standard-dose vitamin D3 group. Safety was assessed according to a list of protocol-specified adverse events of interest by means of participant report and by onset at day 7 of hypercalcemia or severe kidney failure.

### Statistical analysis

Appropriate sample sizes could not be estimated when the trial was planned at the start of the COVID-19 pandemic. Based on data from the literature, in the same context, the expected death rate in the group receiving a standard dose of vitamin D was estimated at 20% [16]. Based on expert opinion, an assumption of 12% reduction in mortality in patients on high-dose vitamin D3 versus standard-dose vitamin D3 was considered (consistent with one previous RCT in ICU reporting a 17% mortality rate reduction in participants who received high-dose vitamin D3 compared to a placebo [17]). So, to demonstrate a difference in mortality between the 2 treatment groups, 125 patients needed to be included in each group to ensure 80% power, while controlling for type I error rate at 5%. To allow for up to 5% nonevaluable or lost to follow-up participants, we set the sample size at 260 participants in total (130 per group), a target that was reached on December 17, 2020.

Outcomes were assessed from the time of randomization. Continuous variables are presented as means and standard deviations (SDs) or medians and interquartile ranges (IQRs), as appropriate, and categorical variables are presented as percentages. Cox proportional hazards models were used to calculate hazard ratios and 95% confidence intervals (CIs) for the comparison of death rates within 14 days in the high-dose and standard-dose vitamin D3 groups after adjustment for randomization strata (i.e., age, oxygen requirement, hospitalization, and use of antibiotics, anti-infective drugs, and/or corticosteroids) as advised previously [18–20], and for baseline imbalances in important prognostic factors (i.e., sex, ongoing cancers, profuse diarrhea, and delirium at baseline) to allow an increase in power and thus conclusions more appropriate to the clinical context [21]. The latter variables were first retained because they appeared unbalanced to the eye in Table 1. Imbalances may be explained by the choice of stratification variables, which was initially made in March to April 2020 when knowledge about COVID-19 was still limited. Since then, additional variables of interest (such as the sex for example) have emerged, which had not been considered initially and thus showed an imbalance here. Kaplan–Meier survival curves were constructed to show cumulative mortality over the 14-day period. As the proportional hazards assumption of the Cox model was questionable according to the graphical approach and to Schoenfeld residuals testing (*P* = 0.09), a landmark approach was also used with day 6 set as the cutoff point of the time window for survival analyses. Analyses were performed on each of the 2-time windows. The same methods were used to analyze the secondary outcomes of the 28-day overall mortality and the 14-day and 28-day mortality due to COVID-19. Finally, exact chi-squared tests were used to examine the between-group differences in the protocol-specified adverse events of interest.

**Table 1:** Baseline characteristics.

	All participants (*n* = 254)	High-dose vitamin D3 group (*n* = 127)	Standard-dose vitamin D3 group (*n* = 127)
**Demographic**			
Median (IQR) age (years)	88 (82 to 92)	87 (81 to 92)	89 (83 to 93)
Female sex	148 (58)	66 (52)*	82 (65)*
Living at home	131 (52)	64 (50)	67 (53)
**Diagnosis of COVID-19**			
Based on SARS-CoV-2 RT-PCR test	240 (95)	121 (95)	119 (94)
Based on chest CT	14 (6)	6 (5)	8 (6)
**COVID-19 worsening risk factor**			
Age ≥75 years	244 (96)	122 (96)	122 (96)
Respiratory support	30 (12)	15 (12)	15 (12)
**Coexisting conditions**			
Ongoing cancer	17 (7)	4 (3)*	13 (10)*
Heart disease	108 (43)	59 (47)	49 (39)
Hypertension	177 (70)	86 (68)	91 (72)
Diabetes	52 (21)	32 (25)	20 (16)
Obesity^†^	43 (22)	21 (21)	22 (24)
Chronic obstructive pulmonary disease	18 (7)	9 (7)	9 (7)
Chronic kidney disease	44 (17)	20 (16)	24 (19)
Chronic liver disease	5 (2)	2 (2)	3 (2)
Major neurocognitive disorder	120 (47)	57 (45)	63 (50)
Cerebrovascular disease	47 (19)	27 (21)	20 (16)
**Median (IQR) number of days since symptom onset**	3 (2 to 4)	3 (2 to 4)	3 (2 to 4)
**WHO Ordinal Scale for Clinical Improvement in COVID-19**			
1. Ambulatory, no limitation of activity	46 (18)	22 (17)	24 (19)
2. Ambulatory, limitation of activity	41 (16)	19 (15)	22 (17)
3. Hospitalized, no oxygen therapy	115 (45)	64 (50)	51 (40)
4. Hospitalized, oxygen therapy (≤4 L/min)	52 (21)	22 (17)	30 (24)
**Relevant symptoms**			
Hyperthermia >38°C^‡^	26 (10)	13 (10)	13 (10)
Delirium	44 (17)	28 (22)*	16 (13)*
Recent fall (<7 days)	27 (11)	15 (12)	12 (9)
Profuse diarrhea	29 (11)	20 (16)*	9 (7)*
Anorexia	70 (30)	40 (32)	37 (29)
Marked asthenia	162 (64)	80 (63)	82 (65)
**Concomitant treatments for COVID-19**			
Hydroxychloroquine	1 (0.4)	1 (1)	0 (0)
Azithromycin	0 (0)	0 (0)	0 (0)
Other antibiotics	62 (24)	31 (24)	31 (24)
Corticosteroids	37 (15)	19 (15)	18 (14)
**Laboratory measures**			
Median (IQR) serum 25(OH)D (nmol/L)^§^	47.8 (26.0 to 78.0)	53.0 (26.0 to 84.0)	43.0 (26.0 to 67.0)
Distribution of serum 25(OH)D^§^			
<25 nmol/L	45 (19)	22 (18)	23 (20)
25 to 50 nmol/L	78 (33)	34 (28)	44 (38)
50 to 75 nmol/L	50 (21)	26 (22)	24 (21)
≥75 nmol/L	65 (27)	39 (32)	26 (22)
Median (IQR) calcium (mmol/L)^§^	2.24 (2.16 to 2.30)	2.24 (2.15 to 2.30)	2.24 (2.17 to 2.30)
Mean (SD) albumin (g/L)^§^	33.7 (5)	33.3 (5)	34.2 (5)
Median (IQR) eGFR (mL/min)^§^	49.0 (37.1 to 63.9)	51.6 (37.8 to 6.0)	47.0 (35.8 to 63.3)
Median (IQR) lymphocytes (giga/L)^§^	1.12 (0.77 to 1.60)	1.12 (0.77 to 1.69)	1.13 (0.80 to 1.57)
Median (IQR) CRP (mg/L)^§^	30.9 (10.0 to 74.0)	31.0 (11.0 to 65.0)	27.5 (8.0 to 84.2)

Values are numbers (percentages) unless stated otherwise. Percentages may not total 100 because of rounding. To convert the values of 25(OH)D to nanograms per milliliter, divide by 2.496.

*Between-group significant differences at baseline using chi-squared test.

^†^Body mass index > 30 kg/m^2^. Data regarding the body mass index were missing for 61 participants.

^‡^Baseline clinical data regarding temperature measurement were missing for 1 participant.

^§^Baseline laboratory data regarding the measures of 25(OH)D were missing for 16 participants, calcium for 9 participants, albumin for 22 participants, eGFR for 27 participants, lymphocytes for 18 participants, and CRP for 21 participants.

COVID-19, Coronavirus Disease 2019; CT, computed tomography; eGFR, estimated glomerular filtration rate; IQR, interquartile range; RT-PCR, reverse transcription polymerase chain reaction; SARS-CoV-2, Severe Acute Respiratory Syndrome Coronavirus 2; SD, standard deviation; 25(OH)D, 25-hydroxyvitamin D.

All *P* values are 2 sided. The full database is held by the trial team, which collected the data from trial sites and performed the analyses at the University Hospital of Angers, France, using SAS, version 9.4, and R, version 3.4.0. The trial was registered on ClinicalTrials.gov on April 14, 2020, NCT04344041.

## Results

### Patients and treatment

**Fig 1:**
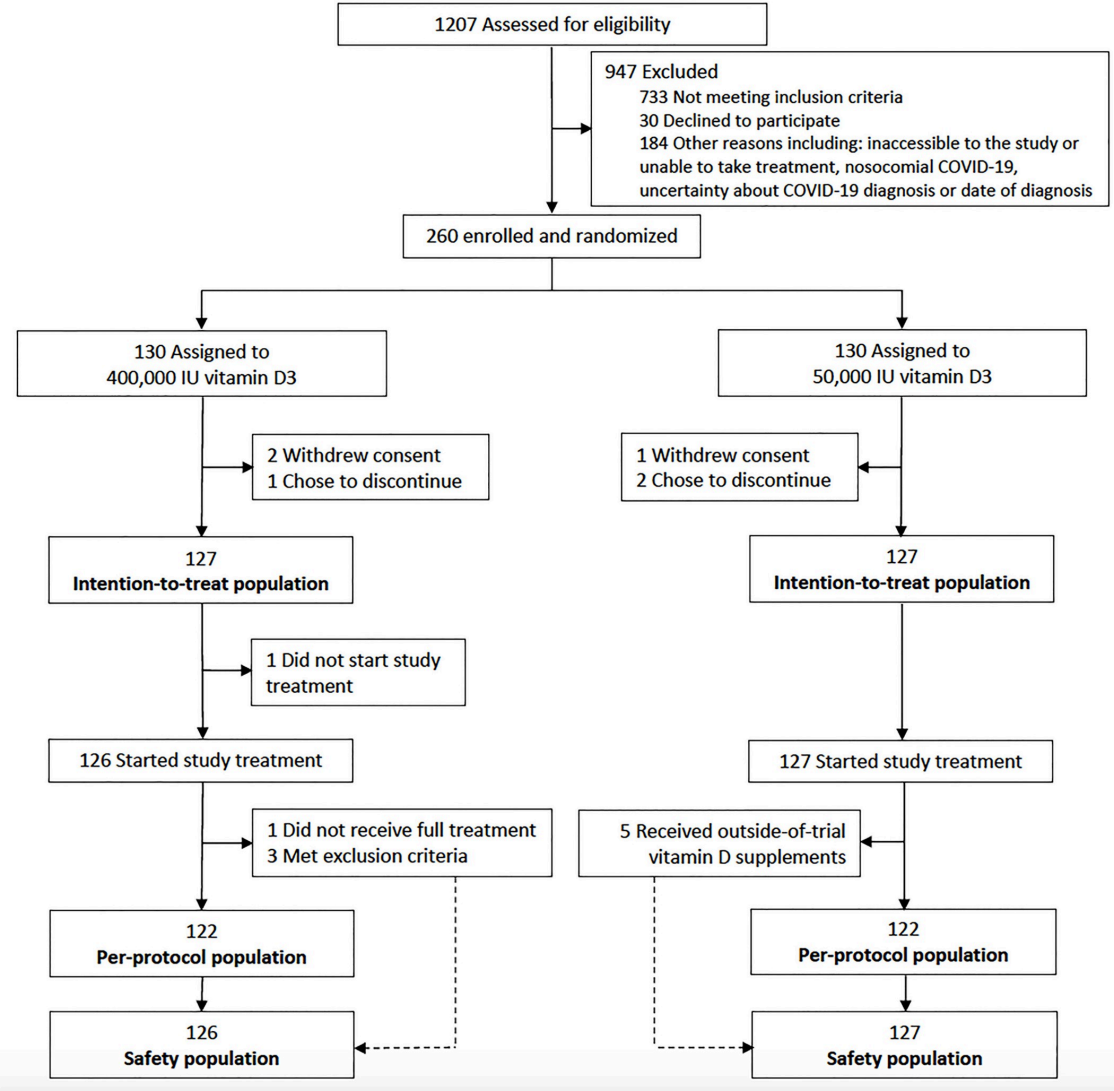
Screening, randomization, and follow-up of the participants in the COVIT-TRIAL study. COVID-19, Coronavirus Disease 2019; COVIT-TRIAL, COvid19 and VITamin d TRIAL.

Between April 15 and December 17, 2020, 1,207 older adults were screened, of whom 260 were deemed eligible (Fig 1). A total of 130 participants were assigned to receive high-dose vitamin D3 and 130 to receive standard-dose vitamin D3. 3 participants in the high-dose group and 3 in the standard-dose group could not be included in the intention-to-treat population because they withdrew their previously written informed consent after randomization or they chose to discontinue the study, so 127 were randomly assigned to receive high-dose vitamin D3 supplementation (intervention) and 127 to receive standard-dose vitamin D3 supplementation (control) (Fig 1). Efficacy analyses were performed on the intention-to-treat population (254 participants). Of note, one participant from the high-dose group who did not receive the study treatment due to immediate death after randomization and 5 participants from the standard-dose group who received outside-of-trial vitamin D supplements were included in the intention-to-treat analysis (Table A in S1 Supplemental Appendix). The per-protocol population involved all participants who met no exclusion criteria, received full study treatment, and were not administered outside-of-trial vitamin D supplements in the standard-dose vitamin D3 group (Table A in S1 Supplemental Appendix). The safety population included all participants who underwent randomization and who received any amount of vitamin D supplement (253 participants).

Participants’ baseline characteristics are shown in Table 1. The median (IQR) age of the participants was 88 (82 to 92) years, and 148 participants (58%) were women. A total of 244 participants (96%) were aged 75years and older (Fig A in S1 Supplemental Appendix). No participants were receiving mechanical ventilation at randomization; 21% were receiving oxygen therapy. There were clinically and statistically significant imbalances in relevant baseline characteristics between the high-dose and standard-dose vitamin D3 groups regarding the proportion of women and of participants with ongoing cancers, delirium, and profuse diarrhea. No imbalances in biological measures were identified, including no difference in serum 25(OH)D concentration at baseline (respectively, 53.0 (26.0 to 84.0) nmol/L in the high-dose group and 43.0 (26.0 to 67.0) nmol/L in the standard-dose group). The serum 25(OH)D concentration achieved at day 7 was 150.5 (117.0 to 196.5) nmol/L in the high-dose vitamin D3 group and 64.5 (43.0 to 85.0) nmol/L in the standard-dose group (*P* < 0.001, Fig B in S1 Supplemental Appendix). Final follow-up was on January 14, 2021. Use of treatments for COVID-19 was similar between the participants allocated high-dose vitamin D3 and those allocated standard-dose vitamin D3. No participants were in the ICU at the time of entering the trial. Corticosteroids were initiated in 34 participants (27%) in the high-dose group and 41 (32%) in the standard-dose group. Two participants were intubated during the conduct of the study (one in each group). A total of 34 participants (27%) received oxygen therapy during the study in the high-dose group and 40 (32%) in the standard-dose group.

### Primary outcome

**Fig 2:**
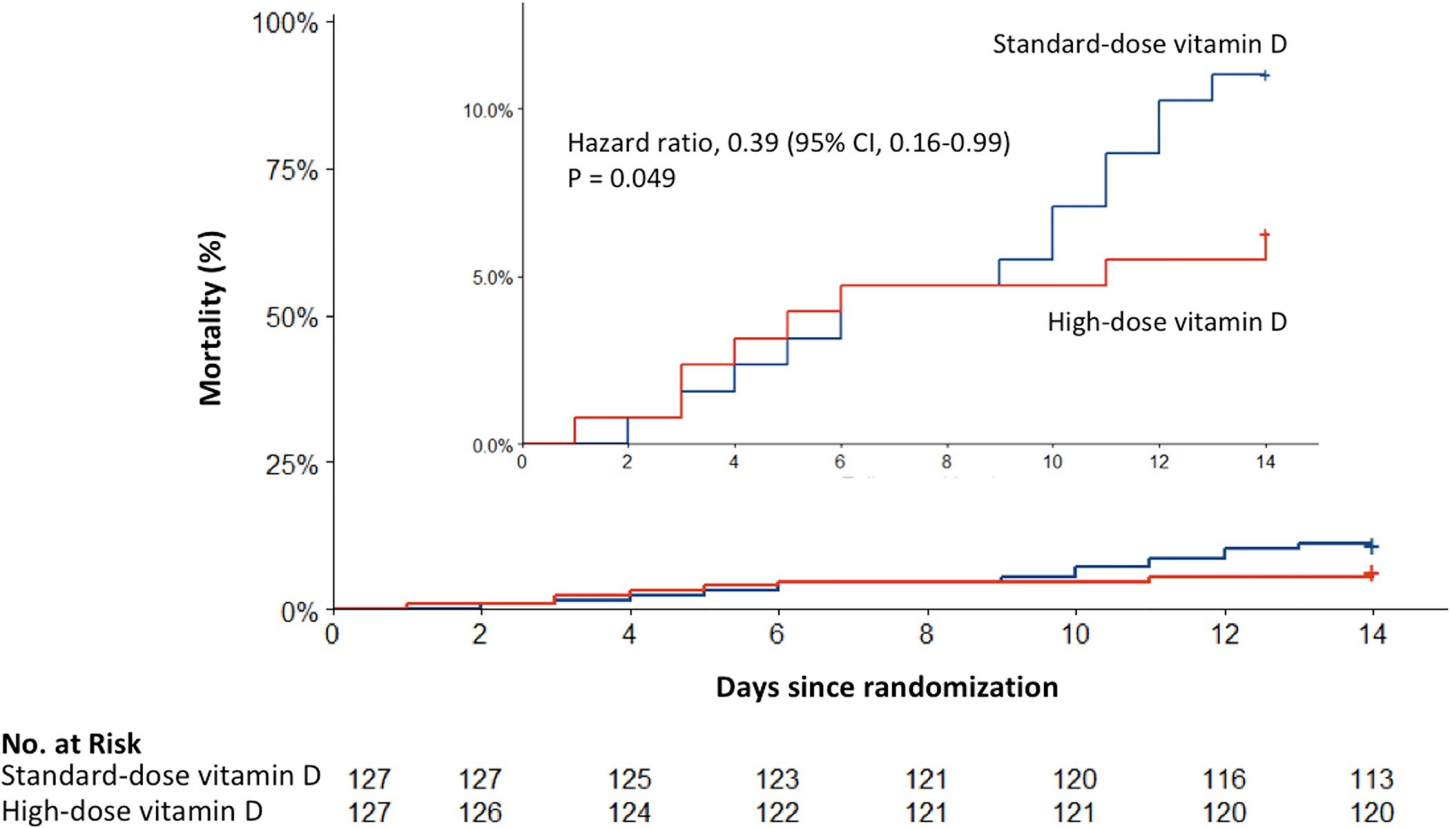
Effect of allocation to high-dose or standard-dose vitamin D3 on 14-day mortality. Overall death at 14 days (the primary outcome) occurred in 8 of 127 patients (6%) in the high-dose vitamin D group and in 14 of 127 patients (11%) in the standard-dose vitamin D group. The insert shows the same data on an expanded y axis. CI, confidence interval.

Death at 14 days (primary outcome) occurred in 8 of 127 participants (6%) in the high-dose vitamin D3 group and in 14 of 127 participants (11%) in the standard-dose vitamin D3 group (unadjusted hazard ratio 0.56 [95% CI, 0.24 to 1.35] *P* = 0.20; adjusted hazard ratio 0.39 [95% CI, 0.16 to 0.99] *P* = 0.049) (Fig 2).

The number needed to treat for one person to benefit (NNTB) was 21 [NNTB 9 to ∞ to number needed to treat for one person to harm (NNTH) 46] (Table 2) (unadjusted hazard ratio 0.49 [95% CI, 0.20 to 1.21] *P* = 0.12; adjusted hazard ratio 0.35 [95% CI, 0.13 to 0.90] *P* = 0.003, and NNTB of 17 [NNTB 8 to ∞ to NNTH 79] in the per-protocol population).

**Table 2:** Effect of allocation to high-dose or standard-dose vitamin D3 supplementation on the primary and secondary outcomes, in intention-to-treat and per-protocol populations.

Outcome	High-dose vitamin D3 supplementation	Standard-dose vitamin D3 supplementation	Relative risk (95% CI) *P* value	Risk difference	Unadjusted hazard ratio (95% CI) *P* value	Adjusted hazard ratio (95% CI) *P* value
	No./total no. (%)			(%)		
	**Intent-to-treat population**					
Primary outcome: 14-day overall mortality	8/127 (6)	14/127 (11)	0.57 (0.25 to 1.32) 0.19	4.7	0.56 (0.24 to 1.35) 0.20	0.39 (0.16 to 0.99) 0.049
Secondary outcome: 28-day overall mortality	19/126 (15)	21/126 (17)	0.91 (0.51 to 1.60) 0.73	1.6	0.89 (0.48 to 1.65) 0.70	0.70 (0.36 to 1.36) 0.29
	**Per-protocol population**					
Primary outcome: 14-day overall mortality	7/122 (6)	14/122 (11)	0.50 (0.21 to 1.20) 0.12	5.7	0.49 (0.20 to 1.21) 0.12	0.35 (0.13 to 0.90) 0.03
Secondary outcome: 28-day overall mortality	17/121 (14)	21/121 (17)	0.81 (0.45 to 1.46) 0.48	3.3	0.78 (0.41 to 1.49) 0.45	0.62 (0.31 to 1.22) 0.17

Intent-to-treat analyses in 127 participants and per-protocol analyses in 122 participants. Data regarding vital status at day 28 were missing for 1 participant in the high-dose vitamin D3 group and 1 participant in the standard-dose vitamin D3 group. Adjusted analyses were controlled for randomization strata (i.e., age, oxygen requirement, hospitalization, and use of antibiotics, anti-infective drugs, and/or corticosteroids) and baseline imbalances in important prognostic factors (i.e., sex, ongoing cancers, profuse diarrhea, and delirium at baseline).

CI, confidence interval.

The effect of high-dose vitamin D3 supplementation on 14-day mortality was independent of baseline 25(OH)D level with a *P* value of 0.83 for the interaction between the randomization arm and the subgroup of participants with baseline vitamin D insufficiency (i.e., 25(OH)D concentration <50 nmoL/L according to the definition of the World Health Organization [22]) compared to the subgroup with baseline 25(OH)D ≥ 50 nmoL/L. The landmark analysis on the first time window (i.e., from the first to the fifth day) of the effect of high-dose versus standard-dose vitamin D3 supplementation found an adjusted hazard ratio of 1.30 [95% CI, 0.31 to 5.35] (*P* = 0.72) for the mortality between day 0 and day 5 and an adjusted hazard ratio of 0.11 [95% CI, 0.02 to 0.52] (*P* = 0.006) for the mortality between day 6 and day 14 (second time window).

### Secondary outcome

By the end of the trial, 40 of 252 participants had died (16%). Death at day 28 occurred in 19 participants in the high-dose vitamin D3 group (15%) and 21 participants (17%) in the standard-dose vitamin D3 group. The unadjusted hazard ratio in the high-dose vitamin D3 group was 0.89 [95% CI, 0.48 to 1.65] (*P* = 0.70), and the adjusted hazard ratio was 0.70 [95% CI, 0.36 to 1.36] (*P* = 0.29) (Fig C in S1 Supplemental Appendix). The landmark analysis on the second time window (i.e., from day 6 to day 28) of the effect of high-dose versus standard-dose vitamin D3 supplementation found an adjusted hazard ratio of 0.54 [95% CI, 0.25 to 1.17] (*P* = 0.12) for the mortality between day 6 and day 28.

### Other clinical outcomes

Most deaths were due to COVID-19, and such deaths were less frequent in the high-dose vitamin D3 group than in the standard-dose vitamin D3 group (Table B in S1 Supplemental Appendix). The adjusted hazard ratio for 14-day mortality due to COVID-19 was 0.33 [95% CI, 0.12 to 0.86] (*P* = 0.02) in the high-dose versus standard-dose group, with a NNTB of 18 [NNTB 8 to ∞ to NNTH 77] (Table C in S1 Supplemental Appendix). Specifically, the adjusted hazard ratio was 1.02 [95% CI, 0.23 to 4.58] (*P* = 0.98) during the first time window between day 0 and day 5 and 0.11 [95% CI, 0.02 to 0.52] (*P* = 0.006) during the second time window between day 6 and day 14. Regarding the 28-day mortality due to COVID-19, the adjusted hazard ratio of high-dose versus standard-dose vitamin D3 supplementation was 0.55 [95% CI, 0.27 to 1.12], *P* = 0.10 (adjusted hazard ratio of 0.48 [95% CI, 0.23 to 0.99] (*P* = 0.047) in the per-protocol population) (Table C in S1 Supplemental Appendix), with an adjusted hazard ratio of 0.43 [95% CI, 0.19 to 0.98] (*P* = 0.045) for the mortality due to COVID-19 during the time window from day 6 to day 28.

Other prespecified analyses, including subgroup analyses comparing the participants according to their changes in serum 25(OH)D concentrations between baseline and day 7, could not be performed due to the limited size of each subgroup with all data available and consequent lack of statistical power to provide interpretable results on these extra outcomes.

### Safety

There were no significant between-group differences in the protocol-specified adverse events of interest (Table 3). Overall, no participants in the high-dose and standard-dose vitamin D3 groups stopped the trial because of an adverse event.

**Table 3:** Protocol-specified adverse events.

	High-dose vitamin D3 group (*n* = 126)	Standard-dose vitamin D3 group (*n* = 127)
At least 1 adverse event—no. of participants (%)	54 (42.9)	44 (34.6)*
Asthenia	21	22
Anorexia	9	14
Nausea and vomiting	6	5
Urinary tract infection	4	5
Accidental fall	4	4
Headache	2	4
Cardiac rhythm or conduction disorders	4	1
Hypercalcemia	3	0
Altered general condition	2	1
Articular pain	2	1
Atrial fibrillation	2	1
Dehydration	2	0
Occlusive syndrome	2	0
Respiratory distress	1	1
Nephrolithiasis	1	1
New-onset severe kidney failure	1	1
Macroscopic hematuria	1	1
Digestive or gastrointestinal bleeding	1	1
Anemia	1	0
Ischemic colitis	1	0
Methicillin-resistant *Staphylococcus aureus* bacteremia	1	0
Conjunctivitis	0	1
Constipation	0	1
Diarrhea	1	0
Pulmonary embolism	1	0
Sudden left hemiparesis	1	0
Hypervitaminosis D	1	0
Hypokalemia	0	1
Iron deficiency	1	0
Increase in anxiety disorders	0	1
Malaise with loss of consciousness	1	0
Malaise without loss of consciousness	0	1
Oral and lingual mycosis	1	0
Edema of the lower limbs	1	0
Prostatitis	1	0
Tremor increase	0	1
Vision disorders	1	0
At least 1 serious adverse event related or possibly related to study treatment—no. of participants (%)	3 (2.4)	0 (0.0)**

One participant in the high-dose vitamin D3 group did not receive the allocated treatment and was excluded from the safety analyses.

*P value for the difference = 0.18 (chi-squared test)

**P value for the difference = 0.12 (Fisher exact test).

## Discussion

In this multicenter open-label RCT, the administration of high-dose versus standard-dose cholecalciferol supplementation to infected older adults within 72 hours after the diagnosis of COVID-19 was associated with reduced overall mortality at day 14. High-dose cholecalciferol was safe and did not result in more frequent adverse effects compared to the standard dose. Some benefits were also found on the 14-day mortality due to COVID-19 as well as on the overall mortality between day 6 and day 14, a critical period in COVID-19, during which inflammatory lung damage is particularly frequent and severe [6]. In contrast, there was no evidence that the single high-dose vitamin D3 administered early in COVID-19 provided any benefit on overall mortality for up to 28 days.

The possibility of a beneficial role of vitamin D supplementation in COVID-19 has been the matter of extensive discussion since the start of the pandemic based on previous meta-analyses of RCT reporting protective effect on respiratory tract infections [23,24]. While our trial was being conducted, 4 other RCT aimed at determining whether vitamin D supplementation improves COVID-19 outcomes have been published. In the SHADE study (India), which randomly assigned 40 middle-aged adults with COVID-19 and vitamin D deficiency to 50,000 IU vitamin D3 per day for 7 days or placebo, the proportion of negative conversion of SARS-COV-2 by 21 days was higher with vitamin D than with placebo (63% versus 21%, *P* = 0.02) [25]. In a RCT conducted in Saudi Arabia among 69 mild-to-moderate COVID-19 middle-aged patients with suboptimal vitamin D status, the assignment to 5,000 IU daily oral vitamin D3 supplementation for 2 weeks reduced the time to recovery for cough (6.2 ± 0.8 days versus 9.1 ± 0.8 days, *P* = 0.04) and ageusia (11.4 ± 1.0 versus 16.9 ± 1.7 days, *P* = 0.035) compared to 1,000 IU daily supplementation [26]. In a RCT conducted in Spain, which randomly assigned 76 middle-aged adults hospitalized for COVID-19 to standard care and oral calcifediol (0.532 mg at baseline followed by 0.266 mg at day 3 and day 7) or standard care alone, the proportion of individuals who needed ICU treatment was lower with calcifediol than in the control group (2% versus 50%, *P* < 0.001) [27]. Finally, a RCT conducted in Brazil, which randomly assigned 240 middle-aged participants hospitalized for moderate-to-severe COVID-19 to 200,000 IU vitamin D3 supplementation or placebo administered 10.3 days after symptoms onset on average, did not find any effect of supplementation on the length of hospital stay [28]. These previous studies did not investigate as a primary outcome the effect of vitamin D supplementation on the survival of patients with COVID-19.

Our trial has several strengths and fundamental differences in design from previous RCT which helped elucidate the potential benefits of vitamin D supplementation on survival in COVID-19. We included older participants at high risk of both vitamin D insufficiency and COVID-19 worsening. All participants received the intervention (except for the participant who died immediately after randomization in the high-dose vitamin D3 group). Overall use of outside-of-trial vitamin D supplements was low. The loading high dose of oral vitamin D3 administered early in the disease resulted from the first week in a large difference in 25(OH)D concentrations between the trial groups. In this regard, the cumulative mortality curve (Fig 2) and the landmark-based predictions suggest that high-dose vitamin D3 supplementation administered early in COVID-19 is unlikely to improve ongoing organic failures and to prevent deaths in the first days of the infection, but is more likely to prevent secondary worsening of COVID-19 related to the uncontrolled inflammatory chain reaction that characterizes the cytokine storm and contributes to inflammatory lung damages and acute respiratory distress syndrome [6,29]. These results are consistent with the well-known anti-inflammatory properties of vitamin D [3] and its ability to regulate the renin–angiotensin system [4,5], which could help curb the cytokine storm and the risk of severe and fatal forms of COVID-19.

The open-label design of the COVIT-TRIAL study represents a risk of bias in the interpretation of the results. Such design was chosen to improve feasibility in accordance with a full-scale validation test [30]. This limitation is probably of very little consequence due to the hard outcome (survival), and we consider it unlikely that the placebo effect of taking open-label high-dose vitamin D3 might have prevented death in COVID-19 patients. Moreover, no influence of the open-label design was expected on the declaration of all-cause mortality at day 14 (primary outcome), which has the merit of being an objective and indisputable outcome. Likewise, the same treatments known to improve survival in COVID-19 were used in both groups during the follow-up, whether regarding corticosteroids, oxygen therapy, or intubation. This absence of interference in care strategies according to the assignment to the trial groups was expected as the efficacy of high-dose vitamin D3 was not demonstrated at the time of the trial.

As a second limitation, it is plausible that our trial was underpowered because of the lack of preliminary data in the early stage of the COVID-19 pandemic that would have allowed an accurate power calculation. Our starting hypothesis expecting a 12% mortality reduction in the high-dose versus standard-dose vitamin D3 group might have been too optimistic, with consequently a greater beta risk. Moreover, it is likely that the power suffered from the fact that the comparator group received a standard dose of vitamin D3 rather than a placebo. However, not supplementing older adults would not have been deemed ethical since this population is particularly at risk of vitamin D insufficiency [22]. Moreover, the use here of covariates adjustment was able to offset this limitation, allowed an increase in statistical power, and, thus, provided conclusions more appropriate to the clinical context [21].

We used very high doses of vitamin D3 in this trial, although 2 previous meta-analyses reported significant efficacy of daily low doses of vitamin D in preventing acute respiratory tract infections [23,24]. Our trial was, however, not aimed at preventing the onset of COVID-19 but at improving survival during the acute phase of the disease. As devised by Binkley and colleagues [31], vitamin D supplementation with physiologic doses to achieve widely accepted 25(OH)D levels considered adequate may not be the same as large pharmacologic doses. In this regard, it is plausible that high-dose vitamin D might have effects as a “drug” that are not observed with “supplementation” doses. Thus, to provide the greatest chance of finding benefit in life-threatening COVID-19, the dose regimen in our trial was designed to result in rapid attainment and maintenance of serum levels that were as high as safely possible [32]. There is concern that 25(OH)D concentrations above 125 nmol/L may be associated with adverse effects [33]. In our trial, the administration of 400,000 IU vitamin D3 resulted in a median 25(OH)D concentration of 150 nmol/L at day 7 without any differences compared to 50,000 IU vitamin D3 regarding the protocol-specified adverse events of interest (Table 3). In line with recent large RCTs that administered for several years 2,000 to 4,000 IU vitamin D3 per day to participants with a very satisfactory baseline vitamin D status [34,35], our study reveals that the risks associated with high-dose vitamin D3 supplementation are minimal during the study period. The clinical implication is that, in the absence of toxicity and given the benefits of high-dose vitamin D on 14-day mortality, a combination therapy with both standard treatments for COVID-19 and high doses of vitamin D3 may be proposed to at-risk older patients with COVID-19 within the first hours of the infection. However, the lack of protection after 28 days questions the single administration of vitamin D3 at the very beginning of the disease. Since the half-life of 25(OH)D is about 2.5 weeks, which implies that 25(OH)D at day 28 would be about half that at day 14, a continuous daily (or weekly) vitamin D supplementation following the loading dose [23] might be required to improve late survival at 28 days, but this deserves further studies especially since the serum 25(OH)D concentrations at day 14 and/or day 28 were not measured here. Similarly, our study was not designed to determine whether vitamin D3 supplementation can help prevent SARS-CoV-2 infection.

In conclusion, we observed in this multicenter, open-label, RCT that the administration of high-dose versus standard-dose vitamin D3 supplementation to older adults within 72 hours after the diagnosis of COVID-19 was associated with reduced overall mortality at day 14. As this protective effect was no longer observed after 28 days, the potential interest of a maintenance vitamin D treatment to sustainably improve survival at day 28 should be addressed in further studies. This simple, safe, and inexpensive treatment may be of interest as an adjuvant to provide a bridge to recovery for at-risk older adults facing the emergence of immune escape variants.

## Supporting information

S1 CONSORT Checklist CONSORT 25-item checklist. CONSORT, Consolidated Standards of Reporting Trials.
(DOC)

S1 Supplemental Appendix Supplemental appendices. Members of the COVIT-TRIAL study group. Fig A: Age distribution of participants included in the COVIT-TRIAL study. Fig B: Violin plots showing (A) the distribution of the serum 25(OH)D concentration (nmol/L) at baseline and day 7 and (B) the distribution of the serum 25(OH)D concentration (nmol/L) at day 7 in the high-dose and standard-dose vitamin D3 groups. Fig C: Time to death according to trial groups in the intention-to-treat and per-protocol populations. Fig D: Mortality due to COVID-19 at 14 days in the intention-to-treat and per-protocol populations. Table A: Major violations to the protocol leading to exclusion of the per-protocol population. Table B: Adjudicated causes of death according to trial groups. Table C: Relative risks and 95% CIs for the mortality due to COVID-19, according to randomized assignment to high-dose or standard-dose vitamin D supplementation, in intention-to-treat and per-protocol populations. Protocol. CI, confidence interval; COVID-19, Coronavirus Disease 2019; COVIT-TRIAL, COvid19 and VITamin d TRIAL; 25(OH)D, 25-hydroxyvitamin D.
(DOCX)

## Abbreviations:

ACE2: angiotensin converting enzyme 2
ANSM: French National Agency for Medicines and Health Products Safety
CI: confidence interval
CONSORT: Consolidated Standards of Reporting Trials
COVID-19: Coronavirus Disease 2019
COVIT-TRIAL: COvid19 and VITamin d TRIAL
CT: computed tomography
ICU: intensive care unit
IQR: interquartile range
NNTB: number needed to treat for one person to benefit
NNTH: number needed to treat for one person to harm
PaO2/FiO2: partial pressure of oxygen in arterial blood/fraction of inspired oxygen ratio
RCT: randomized controlled trial
RT-PCR: reverse transcription polymerase chain reaction
SARS-CoV-2: Severe Acute Respiratory Syndrome Coronavirus 2
SD: standard deviation; 25(OH)D, 25-hydroxyvitamin D
SpO2: peripheral capillary oxygen saturation
