# Supplementary material for: High-dose versus standard-dose vitamin D supplementation in older adults with COVID-19 (COVIT-TRIAL): A multicenter, open-label, randomized controlled superiority trial
Source: PLoS Med. 2022 May 31;19(5):e1003999. doi: 10.1371/journal.pmed.1003999 (PMC9154122; doi:10.1371/journal.pmed.1003999)
Supplement: S1 Supplemental Appendix — Members of the COVIT-TRIAL study group. Fig A: Age distribution of participants included in the COVIT-TRIAL study. Fig B: Violin plots showing (A) the distribution of the serum 25(OH)D concentration (nmol/L) at baseline and day 7 and (B) the distribution of the serum 25(OH)D concentration (nmol/L) at day 7 in the high-dose and standard-dose vitamin D3 groups. Fig C: Time to death according to trial groups in the intention-to-treat and per-protocol populations. Fig D: Mortality due to COVID-19 at 14 days in the intention-to-treat and per-protocol populations. Table A: Major violations to the protocol leading to exclusion of the per-protocol population. Table B: Adjudicated causes of death according to trial groups. Table C: Relative risks and 95% CIs for the mortality due to COVID-19, according to randomized assignment to high-dose or standard-dose vitamin D supplementation, in intention-to-treat and per-protocol populations. Protocol. CI, confidence interval; COVID-19, Coronavirus Disease 2019; COVIT-TRIAL, COvid19 and VITamin d TRIAL; 25(OH)D, 25-hydroxyvitamin D. (DOCX) [file pmed.1003999.s002.docx]

**Supplementary Appendix**

This appendix has been provided by the authors to give readers additional information about their work.

# Content

**Members of the COVIT-TRIAL Study Group**..…………………………………….……………..page 2

**Supplementary Figures**……………………………..….…………………………………………....page 3

**Figure A.** Age distribution of participants included in the COVIT-TRIAL study…………….…......page 4

**Figure B.** Figure S2. Violin plots showing (A) the distribution of the serum 25-hydroxyvitamin D concentration (nmol/L) at baseline and day 7, and (B) the distribution of the serum 25-hydroxyvitamin D concentration (nmol/L) at day 7 in the high-dose and standard-dose vitamin D3 groups..…….…….page 5

**Figure C.** Time to death according to trial groups in the intention-to-treat and per-protocol populations……………………………………………...………………………………………….....page 6

**Figure D.** Mortality due to COVID-19 at 14 days in the intention-to-treat and per-protocol populations……...………………………………………………………………………………….....page 7

**Supplementary Tables**. ….……………………………………………………………………….....page 8

## Table A. Major violations to the protocol leading to exclusion of the per-protocol population..........page 9

**Table B.** Adjudicated causes of death according to trial groups……………………….…….……..page 10

## Table C. Relative risks and 95% confidence intervals for the mortality due to COVID-19, according to randomized assignment to high-dose or standard-dose vitamin D supplementation, in intention-to-treat and per-protocol populations.……………..….…………...…………………………………………page 11

**Protocol**……………………..………………………….……………………………………………page 12

# **Members of the COVIT-TRIAL Study Group**

**COVIT-TRIAL Steering Committee**:

Cédric Annweiler (Chair), Mélinda Beaudenon, Loïc Carballido, Astrid Darsonval, Justine Gonsard, Elsa Parot-Schinkel (all at the University Hospital of Angers, France)

**Scientific Consultant**:

Jean-Claude Souberbielle (Assistance Publique – Hôpitaux de Paris, France).

**Other Members of the COVIT-TRIAL Research Group:**

(Angers University Hospital): Marine Asfar, Jean Barré, Florian Berteau, Sophie Boucher, Antoine Brangier, Olivier Brière, Lucie Boucard, Jean-Marie Chrétien, Mathieu Corvaisier, Philippe Codron, Marine De La Chapelle, Valérie Daniel, Astrid Darsonval, Robin Dhersin, Vincent Dubée, Elise Dugleux, Guillaume Duval, Jennifer Gautier, Chrystelle Gilet, Amélie Goubaud, Mialy Guenet, Catherine Hue, Cécile Jaglin, Aurélie Jamet, Denise Jolivot, Carole Lacout, Maxime Le Floch, Pierre-Marie Le Meur, Sébastien Lleonart, Jocelyne Loison, Rafaël Mahieu, Romain Ordonez, Marie Otekpo, Virginie Pichon, Valérie Rabier, Laëtitia Rault, Sami Rehaiem, Jérémie Riou, Hélène Rivière, Romain Simon, Karine Stenvot, Marine Thomas, Wojciech Trzepizur, Yves-Marie Vandamme, Amandine Vildy (Bordeaux University Hospital): Guillaume Albaret, Isabelle Bourdel-Marchasson, Aurélie Cherchouly, Emilie Clabé, Marie Danet-Lamasou, Jessica Durrieu, Paul Gassie, Aurélie Lafargue, Claire Langlade, Marion Lartigau, Fara Ratsimbazafy, Claire Roubaud-Baudron, Benjamin Sourisseau (Le Mans Hospital): Florence Dangeul-Potier, Céline Lagrange, Pierre Ménager, Catherine Naveau, Frédéric Scholastique (Limoges University Hospital): Sophie Boyer, Noëlle Cardinaud, Estelle Champagne, Edouard Desvaux, Hélène Fortes-Beacco, Caroline Gayot, Nicolas Giroult, Cécile Laubarie-Mouret, Thai Binh Nguyen, Arnaud Papon, Chloé Radu-Dubois, Françoise Renon-Carron, Achille Tchalla (Nantes University Hospital): Carole Agasse, Gilles Berrut, Anne Sophie Boureau, Céline Brouessard, Marie Laure Bureau, Guillaume Chapelet, Laure de Decker, Pauline Gendre, Pamela Hublain, Sylvain Le Gentil, Marie Mathieu, Agnès Rouaud, Etienne Seronie-Doutriaux (Nice University Hospital): Justine Bellegarde, Christelle Boczek, Nihed Boughdiri, Sophie Breaud, Michel Carles, David Chirio, Johan Courjon, Eric Cua, Alexiane Decorbez, Elisa Demonchy, Olivier Guérin, Ramona Levinschi, Guillaume Marrane, Anthony Mauclere, Emeline Michel, Virginie Piovano, Gary Pommier, Karine Risso, Guillaume Sacco, Irit Touitou (Saint-Etienne University Hospital): Fahima Benkhider, Gaëlle Bertoletti, Elisabeth Botelho-Nevers, Nadine Casimir, Karine Castro-Lionard, Thomas Celarier, Maxime Courtial, Monique D’Hautefeuille, Anne-Cécile Gallo-Blandin, Sophie Garnon, Ludovic Lafaie, Jérémy Mangavelle, Laure Martinez, Gwénaël Monnier, Albert Mvondo, Cyrille Renaud (Saumur Hospital): Marjorie Houvet, Amal Lisfi (Tours University Hospital): Amal Aidoud, Alexandra Audemard-Verger, Hélène Bansard, Adrien Bigot, Stéphanie Bonte, Lysiane Brick, Maeva Dieu, Nina Duret-Aupy, Abderrahim El Houfia, Bertrand Fougère, Régis Hankard, Stéphanie Jobard, François Maillot, Fanny Poitau, Marie Capucine Trousset.

**COVIT-TRIAL Writing Committee**:

Cédric Annweiler, Mélinda Beaudenon, Sophie Boucher, Thomas Celarier, Guillaume Chapelet, Astrid Darsonval, Bertrand Fougère, Jennifer Gautier, Justine Gonsard, Olivier Guérin, Marjorie Houvet, Pierre Ménager, Elsa Parot-Schinkel, Jérémie Riou, Claire Roubaud-Baudron, Jean-Claude Souberbielle, Achille Tchalla

All the authors vouch for the accuracy and completeness of the data and for the fidelity of the trial to the protocol. The statistical team at the coordinating center analyzed the data and vouches for its accuracy. The first draft of the manuscript was drafted by the first author and approved by all members of the trial steering and writing committees. No one who is not an author contributed to the writing of the manuscript.

**Data and Safety Monitoring Board**

(Voting Members): Sylvie Bonin-Guillaume, MD, PhD (Marseille University Hospital, France); François R Herrmann, MD, MPH (Geneva University Hospitals, Switzerland); Anne-Marie Schott, MD, PhD (Lyon University Hospital, France)

# **Supplementary Figures**

**
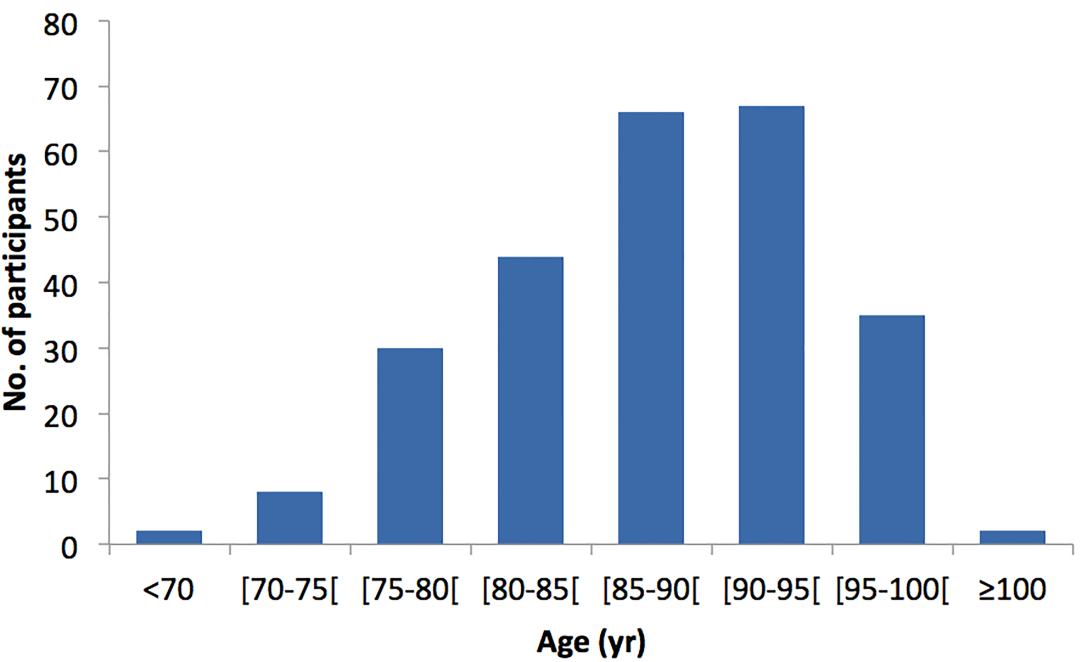
**

**Figure A. Age distribution of participants included in the COVIT-TRIAL study (n = 254)**

**(a)**


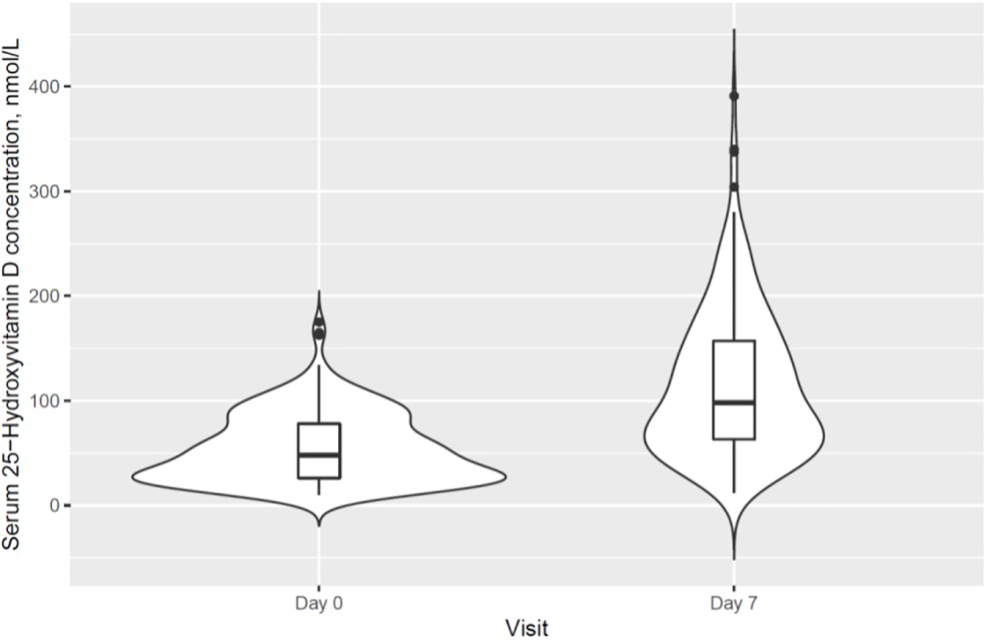


**(b)**

**
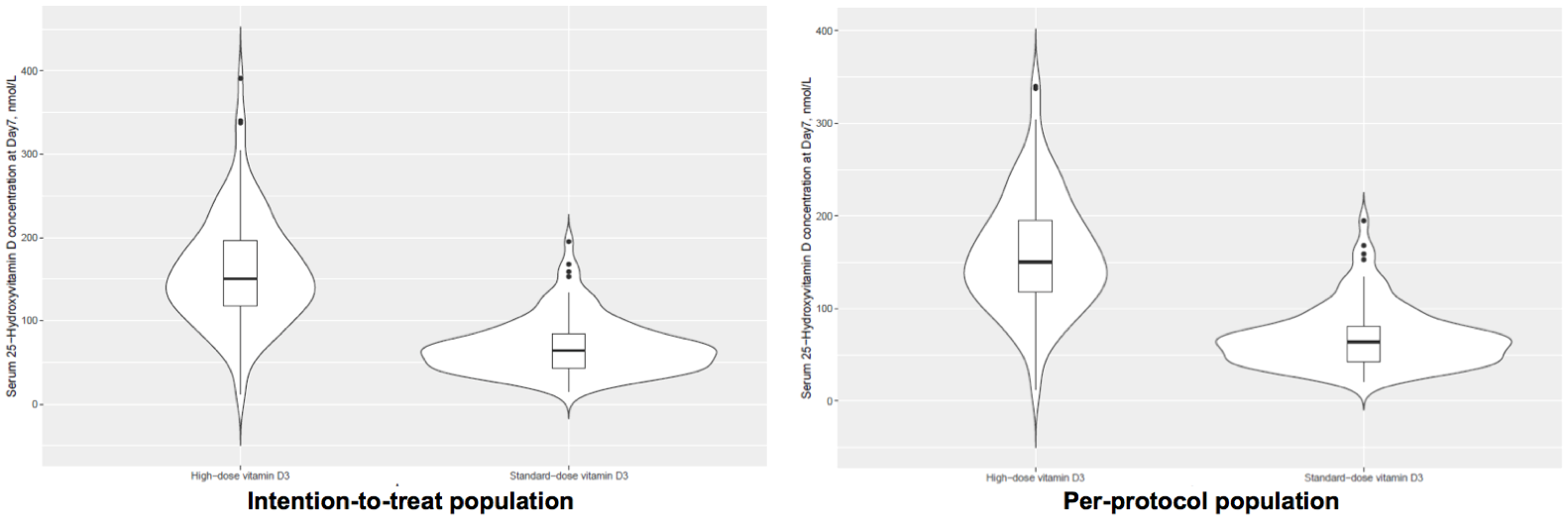
**

**Figure B. Violin plots showing (a) the distribution of the serum 25-hydroxyvitamin D concentration (nmol/L) at baseline and day 7, and (b) the distribution of the serum 25-hydroxyvitamin D concentration (nmol/L) at day 7 in the high-dose and standard-dose vitamin D3 groups.***

Serum 25-hydroxyvitamin D concentration was measured during the baseline and day 7 visits only. Data regarding the measures of 25-hydroxyvitamin D concentration were missing in 16 participants at baseline, and in 40 participants at day 7. To convert the values of 25-hydroxyvitamin D to nanograms per milliliter, divide by 2.496.

In the intent-to-treat population, the median (interquartile range) serum 25-hydroxyvitamin D was 53 (26-84) nmol/L at baseline in the high-dose vitamin D3 group, and 43 (26-67) nmol/L in the standard-dose group; it achieved 151 (117-197) nmol/L at day 7 in the high-dose group, and 65 (43-85) nmol/L in the standard-dose group. In the per-protocol population, the median 25-hydroxyvitamin D was 52 (26-81) nmol/L at baseline in the high-dose vitamin D3 group, and 42 (26-66) nmol/L in the standard-dose group; it achieved 151 (117-196) nmol/L at day 7 in the high-dose group, and 64 (43-81) nmol/L in the standard-dose group. The two graphs in Fig. Bb were superimposable.

* This plot aims to compare the distribution of both variables as a function of visit. The boxes indicate the 75^th^ percentile (upper horizontal line), median (black bold horizontal line), and 25^th^ percentile (lower horizontal line) of the distribution. Surrounding the boxes on each side is a rotated kernel density plot.

**(a)**


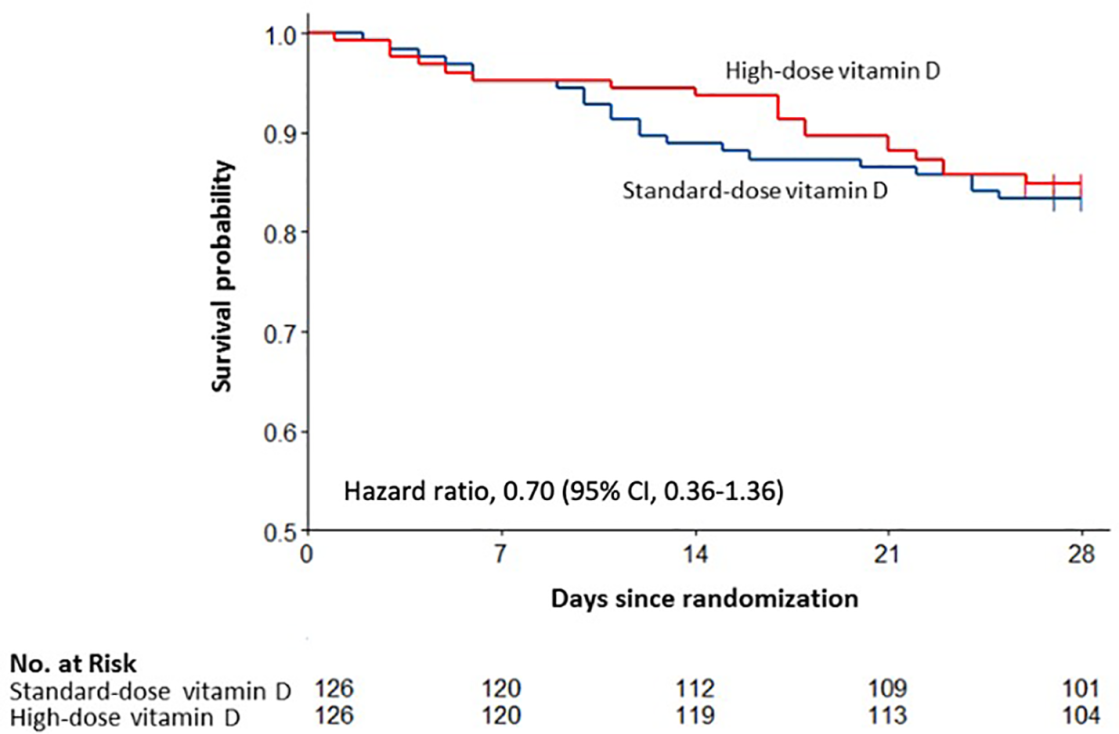


**(b)**


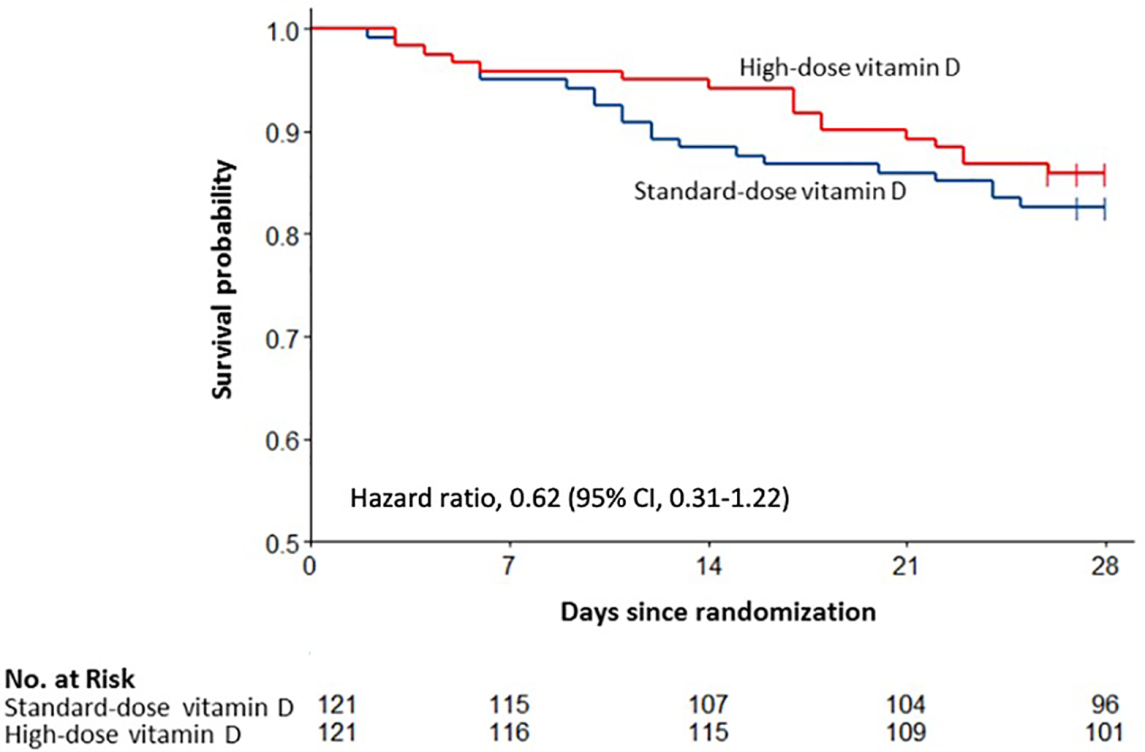


## Figure C. Time to death according to trial groups in the (a) intention-to-treat population (n = 252) and (b) per-protocol population (n = 242).*

## Death at 28 days (the secondary outcome) occurred in 19 of 126 participants (15%) in the high-dose vitamin D group and in 21 of 126 participants (17%) in the standard-dose vitamin D group.

* Shown are Kaplan–Meier estimates of the time from the intervention (administration of high-dose or standard-dose vitamin D supplementation) to the death. Data on vital status at day 28 were missing for one participant in the high-dose vitamin D group and one participant in the standard-dose vitamin D group.

**(a)**


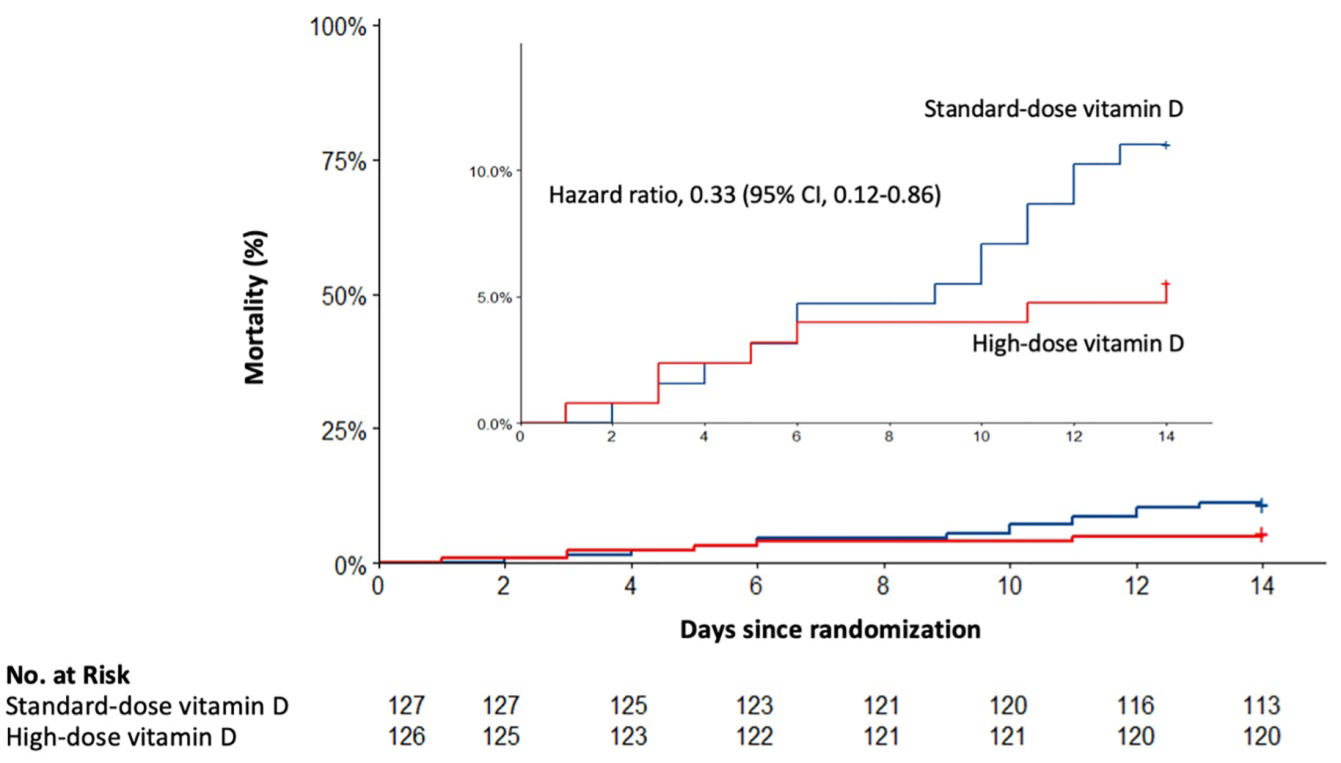


**(b)**

##
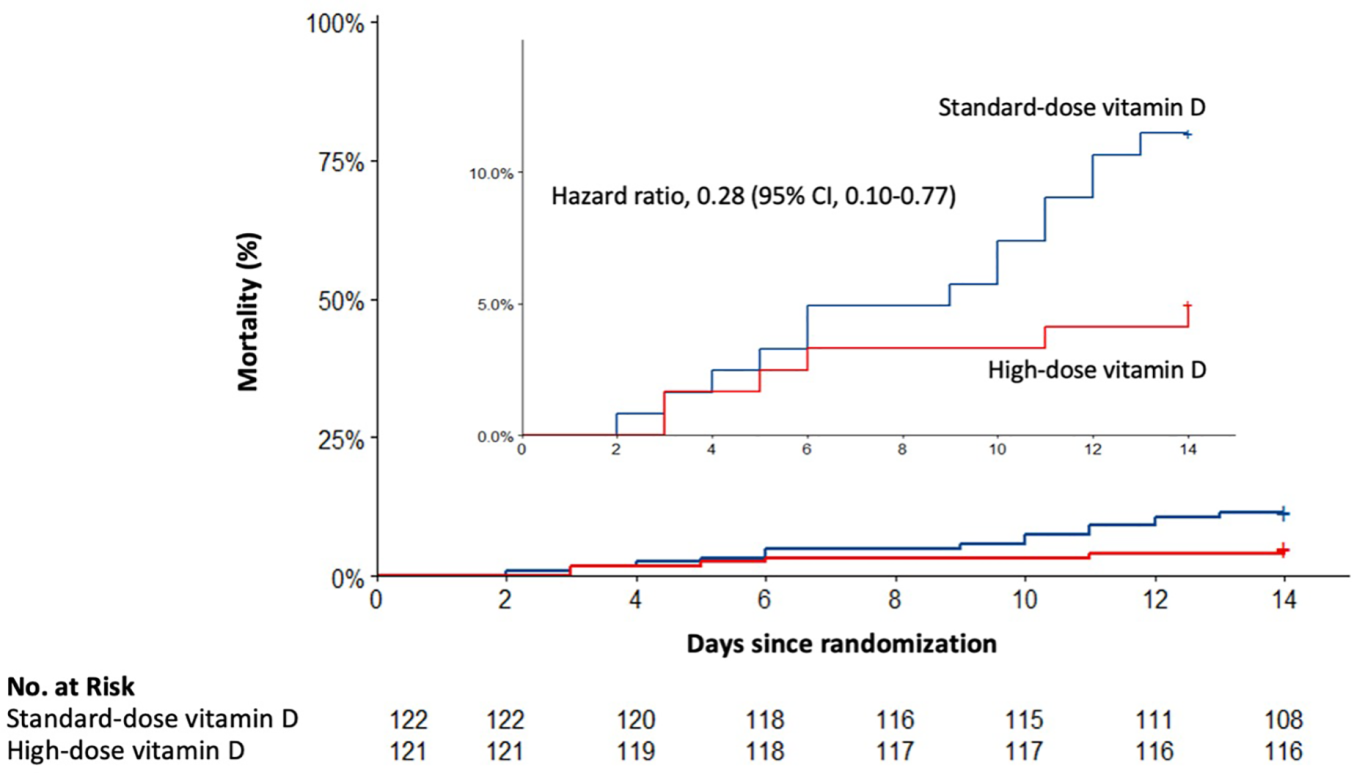


**Figure D. Mortality due to COVID-19 at 14 days in the (a) intention-to-treat population and (b) per-protocol population.**

## Death due to COVID-19 at 14 days occurred in 7 of 126 participants (6%) in the high-dose vitamin D group and in 14 of 127 participants (11%) in the standard-dose vitamin D group. The insert shows the same data on an expanded y axis.

# **Supplementary Tables**

## Table A. Major violations to the protocol leading to exclusion of the per-protocol population.

| **Major violations** | **High-dose**  **vitamin D3 group**  (n=127) | **Standard-dose vitamin D3 group**  (n=127) |
| --- | --- | --- |
| Inclusion and non-inclusion criteria |  |  |
| Presence of at least one non-inclusion criteria | 3 | 0 |
| Deviation in treatment administration protocol |  |  |
| Allocated treatment not started | 1 | 0 |
| No adherence to the allocated treatment (50% of the planned dose administered) | 1 | 0 |
| Administration of outside-of-trial vitamin D supplements to participants in the standard-dose vitamin D group | NA | 5 |

NA=not applicable.

**Table B. Adjudicated causes of death according to trial groups**

| **Adjudicated cause of death** | **no./total no. (%)** | |
| --- | --- | --- |
|  | **High-dose**  **vitamin D3 group**  (n=19) | **Standard-dose**  **vitamin D3 group**  (n=21) |
| COVID-19 | 16 (84) | 21 (100) |
| Acute pulmonary edema | 1 | 0 |
| Digestive bleeding | 1 | 0 |
| Urinary sepsis | 1 | 0 |

## Table C. Effect of allocation to high-dose or standard-dose vitamin D3 supplementation on mortality due to COVID-19, in intention-to-treat and per-protocol populations.*

| **Outcome** | **High-dose**  **vitamin D3 supplementation** | **Standard-dose vitamin D3 supplementation** | **Relative risk (95% CI)**  ***P-value*** | **Risk Difference** | **Unadjusted hazard ratio**  **(95% CI)**  ***P-value*** | **Adjusted hazard ratio**  **(95% CI)**  ***P-value*** |
| --- | --- | --- | --- | --- | --- | --- |
|  | **no./total no. (%)** | |  | **(%)** |  |  |
|  | **Intent-to-treat population** | | | | | |
| 14-day mortality due to COVID-19† | 7/126 (6) | 14/127 (11) | 0.50 (0.21-1.21)  *0.12* | 5.4 | 0.49 (0.20-1.23)  *0.13* | 0.33 (0.12-0.86)  *0.02* |
| 28-day mortality due to COVID-19‡ | 16/123 (13) | 21/126 (17) | 0.78 (0.43-1.42)  *0.42* | 3.7 | 0.76 (0.40-1.45)  *0.40* | 0.55 (0.27-1.12)  *0.10* |
|  | **Per-protocol population** | | | | | |
| 14-day mortality due to COVID-19† | 6/121 (5) | 14/122 (11) | 0.43 (0.17-1.09)  *0.07* | 6.5 | 0.42 (0.16-1.09)  *0.08* | 0.28 (0.10-0.77)  *0.013* |
| 28-day mortality due to COVID-19‡ | 14/118 (12) | 21/121 (17) | 0.68 (0.37-1.28)  *0.23* | 5.5 | 0.66 (0.33-1.29)  *0.22* | 0.48 (0.23-0.99)  *0.047* |

*Data regarding vital status at day 28 were missing for 1 participant in the high-dose vitamin D group and 1 participant in the standard-dose vitamin D group. Adjusted analyses were controlled for randomisation strata (ie, age, oxygen requirement, hospitalization, and use of antibiotics, anti-infective drugs and/or corticosteroids) and baseline imbalances in important prognostic factors (ie, sex, ongoing cancers, profuse diarrhea and delirium at baseline). CI=confidence interval.

†Excluding one death due to another reason than COVID-19

‡Excluding three deaths due to other reasons than COVID-19

**Protocol S1.**

See the following reference (noted as Reference 15 in the manuscript): Annweiler C, Beaudenon M, Gautier J, et al. COVID-19 and high-dose VITamin D supplementation TRIAL in high-risk older patients (COVIT-TRIAL): study protocol for a randomized controlled trial. Trials. 2020;21:1031.
